# Factors Related to Mental Health During the COVID-19 Lockdown in Spain

**DOI:** 10.3389/fpsyg.2021.715792

**Published:** 2021-08-24

**Authors:** Ruth Pinedo, Isabel Vicario-Molina, Eva González Ortega, Andrés Palacios Picos

**Affiliations:** ^1^Department of Psychology, University of Valladolid, Segovia, Spain; ^2^Department of Developmental and Educational Psychology, University of Salamanca, Salamanca, Spain

**Keywords:** COVID-19, lockdown 2020, mental health, risk factors, protective factors, loneliness

## Abstract

The COVID-19 disease has forced governments to adopt exceptional measures. The lockdown decreed in Spain in 2020 required citizens to stay confined at home, which might have affected their mental health. The objective is to identify factors that influenced adults' mental health during this period. A sample of 3,508 adults from the Spanish general population completed an online survey that collected sociodemographic data and information about daily planning and activities, healthy habits, loneliness, coping humor and mental health. Data were analyzed using Structural Equation Modeling. According to the results, the proposed model showed good fit values, and latent variables explained 30% of the variance in mental health. Loneliness, coping humor, healthy habits, age and gender had a significant weight in the prediction of mental health during lockdown. Area of residence, number of days of confinement and number of people in the household were not related to mental health. This study addresses the effect of COVID-19 and social distancing measures by identifying risk and protective factors for the development of mental health difficulties. There is a need to target specific and early interventions aimed at mitigating the psychological impact of the pandemic while increasing well-being, especially in more vulnerable groups.

## Introduction

As a consequence of the COVID-19 pandemic, countries implemented measures to control movement and reduce contact among people. Spain decreed the state of alarm for the management of the health crisis situation which implied a strict stay-at-home lockdown on March 15, 2020 that lasted until June 21, 2020 (Royal Decree, 2020). Private and public meetings, movement and use of public spaces were legislated and enforced by police.

During this quarantine period, the main containment measures affecting the general population were the following:

- The freedom of movement of persons in the public realm was restricted to ensure social distancing; citizens were required to remain confined in their homes except for a very limited number of essential activities that had to be performed individually (except in strictly necessary and duly justified cases): purchasing food items, pharmaceutical products and basic necessities; attending health centers and services; traveling to their place of work; returning to their place of residence; caring for especially vulnerable people; visiting banks; force majeure or critical situations.
- Classroom-based educational activity was suspended in all centers and at all levels, and maintained through the online modalities.
- The opening to the public of the commercial, cultural and recreational activity was suspended, with some exceptions (e.g., food, beverages, pharmaceutical and hygienic products, fuel).
- Attendance at places of worship and civil and religious ceremonies, including funerals, was conditional on the adoption of organizational measures aimed at maintaining a safe distance.
- There was a reduction of operations for certain modes of transport and the obligation to put on sale one third of the available seats to guarantee adequate distance between travelers.
- Borders were closed, and non-essential travel from third countries to the European Union was restricted.

On May 11, 2020, with the start of the official period of “de-escalation,” the lockdown measures decreed in Spain began to relax at different speeds in the different Autonomous Communities and for the different productive sectors (Mateos et al., 2020). In any case, they were one of the most severe in Europe (Rodríguez-González et al., 2020; European Centre for Disease Prevention Control, 2021; Pouso et al., 2021; University of Oxford, 2021; World Health Organization Regional Office for Europe, 2021a), since COVID-19 hit this country particularly hard (World Health Organization Regional Office for Europe, 2021b).

Lockdown and social distancing measures adopted in many countries during the COVID-19 crisis have produced positive outcomes, such as the reduction of infections. However, these measures have been a difficult and unpleasant experience for many people that may have impacted their mental health (Brooks et al., 2020; Holttum, 2020; Losada-Baltar et al., 2020; Osimo, Aiello, Gentili, Ionta and Cecchetto, 2021; Pouso et al., 2021). This situation may have been especially challenging in Spain, where there is a tendency to physical proximity and greetings involving contact between hands, faces and bodies, unlike other countries where people usually maintain an interpersonal distance of more than 1.5 meters (Otero-Iglesias and Molina, 2020).

According to recent reviews, studies examining the psychological and mental impact of the COVID-19 pandemic on the general population—mostly conducted in China—have shown a pooled prevalence of stress, anxiety and depression of about 30% (Salari et al., 2020; Xiong et al., 2020). Hence, it is important to conduct research aiming to understand the psychological impact of COVID-19 in order to design resources to ameliorate the social and psychological effects that pandemics can generate (Saltzman et al., 2020). As in previous pandemics, it is necessary to address the mental health needs of citizens (Lau et al., 2005) and gain evidence about the associated risk and protective factors in order to be prepared and to prevent negative outcomes.

As regards sociodemographic factors, previous studies have generally found lower levels of mental health among women (Barzilay et al., 2020; El-Zoghby et al., 2020; González-Sanguino et al., 2020; Parrado-González and León-Jariego, 2020; Rodríguez-Rey et al., 2020; Xiong et al., 2020) and younger people (Becerra-García et al., 2020; Caballero-Domínguez et al., 2020; El-Zoghby et al., 2020; González-Sanguino et al., 2020; Parrado-González and León-Jariego, 2020; Rodríguez-Rey et al., 2020; Xiong et al., 2020). A smaller proportion of studies found no significant differences according to gender (Becerra-García et al., 2020; Caballero-Domínguez et al., 2020; Lu et al., 2020) or age (Lu et al., 2020; Wang et al., 2020a).

In addition, some previous results indicate that living alone (Becerra-García et al., 2020), in a house with fewer square meters per person (Parrado-González and León-Jariego, 2020), and in an urban environment (El-Zoghby et al., 2020) are related to higher levels of mental health symptoms, while other data suggest the absence of significant differences according to the size of the house (Wang et al., 2020a) or the environment/area in which it is located (Lu et al., 2020). These inconsistencies might be partly explained by the different instruments and concepts used to assess mental health. It should also be noted that some studies provide information about the sociodemographic characteristics of the sample, but do not assess their association to mental health variables (e.g., Yildirim and Güler, 2020).

Finally, some studies (Twenge and Joiner, 2020) have found small differential trends in mental health problems over the course of time during the pandemic (April–May 2020), with a slight lessening in the prevalence of anxiety and an increase in the prevalence of depression, although both symptoms remained high. Similarly, other authors have observed a significant longitudinal reduction in PTSD symptoms after four weeks, although not clinically significant (symptoms remained above the cut-off scores), as well as no significant longitudinal changes in stress, anxiety and depression level (Wang et al., 2020b). Furthermore, individuals with personal characteristics such as emotional stability, resilience and lower levels of alexithymia seem to deal better with lockdown measures throughout the confinement period (Osimo, Aiello, Gentili, Ionta and Cecchetto, 2021).

In such adverse situations, in which social support is more necessary than ever (Usher et al., 2020) and it is difficult to maintain the level or quality of desired social contact, the perception of loneliness may have a greater impact on mental health. Perlman and Peplau (1981) defined loneliness as “the unpleasant experience that occurs when a person's network of social relations is deficient in some important way, either quantitative or qualitative” (p. 31). Thus, loneliness arises from a deficit in personal relationships, and is a subjective phenomenon and a distressing experience. de Jong Gierveld (1998) broadened the definition by recognizing that the origin of loneliness may result from a reduced number of relationships, as well as a lower level of intimacy than what is desired. Other authors have also recognized the importance of the qualitative dimension and cognitive appraisal in the perception of loneliness (e.g., Cacioppo and Cacioppo, 2018). However, loneliness has been recognized only recently as a severe public health issue which could be affecting a third of the people in developed countries (Richard et al., 2017; Cacioppo and Cacioppo, 2018; Ercole and Parr, 2020).

Several studies have documented that perceived loneliness is related to physical and psychological health (e.g., de Jong Gierveld, 1998; Patterson and Veenstra, 2010; Steptoe et al., 2013; Richard et al., 2017) and also to mortality (Holt-Lunstad et al., 2015; Christensen et al., 2020). Thus, a systematic review by Leigh-Hunt et al. (2017) found strong evidence that loneliness and social isolation were associated with an increased risk of premature mortality (up to 26%). Causal effects are difficult to determine, but the relationship could be mediated by mental health (Leigh-Hunt et al., 2017). Likewise, a systematic review by Wang and colleagues pointed out that loneliness and perception of low social support predicted some mental health problems (Wang et al., 2018). Specifically, a number of studies found that loneliness was related to anxiety (Beutel et al., 2017; van Beljouw et al., 2020), depression (Mahon et al., 2006; Adam et al., 2011; Lasgaard et al., 2011; van Beljouw et al., 2020), psychological distress (Richard et al., 2017), suicidal ideation (Beutel et al., 2017) and use of psychotropic drugs (Bekhet and Zauszniewski, 2012). Moreover, loneliness appears to be a risk factor for cognitive health, since feeling lonely has been related to clinical dementia (Holwerda et al., 2014) and cognitive decline over time (Donovan et al., 2017).

During the current pandemic, loneliness could be especially relevant to understand mental health outcomes. A study by Killgore et al. (2020) found that during the stay-at-home orders decreed in the majority of states in the U.S., 43% of the sample exceeded the cut-off for a high level of loneliness, and loneliness was related to depression and suicidal ideation. Although loneliness can be experienced at any developmental stage, the relationship between loneliness and age follows a U-shaped distribution, and high-risk age groups are people under 25 and over 65 years old (Victor and Yang, 2012; Richard et al., 2017). Thus, social distancing measures may have negative effects on the mental health of young people (Beam and Kim, 2020; Chen et al., 2020) and older adults (American Psychological Association Committee on Aging, 2020; Monahan et al., 2020; Roychowdhury, 2020). Among youth, the stress about the pandemic combined with normative stressors (Shanahan et al., 2020) may have negatively affected psychological adjustment, resulting in increased loneliness and depression during the COVID-19 crisis (Ellis et al., 2020). On the other hand, the social isolation measures may especially have affected old adults, an at-risk population (Berg-Weger and Morley, 2020; Vahia et al., 2020). The loss of daily contact with family and friends (Banskota et al., 2020) may exacerbate the consequences of loneliness (Beam and Kim, 2020), reduce the benefits of social and daily care interactions (Monahan et al., 2020) and enhance other vulnerabilities of the population (Roychowdhury, 2020). Nevertheless, as results obtained by Luchetti et al. (2020) showed, social restriction measures do not necessarily increase the perception of loneliness by a large degree.

Likewise, the perception of support and social connection during the pandemic might have benefits on mental health, not only through the reduction of negative symptomatology but also by facilitating adaptation (Saltzman et al., 2020). Thus, Xiao et al. (2020) found that the perception of belonging, trust and social participation during a period of self-isolation at home was related to levels of anxiety, stress and sleep quality.

On the other hand, some factors might alleviate the negative consequences of pandemic-related stress and predict a good adjustment. Coping strategies are responses that effectively reduce the burden of life events and increase psychological well-being (Snyder, 1999). Generally, two ways of coping are distinguished: (a) problem-focused coping, which includes attempts to manage the stressful events (e.g., planning, active coping), and (b) emotion-focused coping, which considers efforts to reduce the emotional outcomes of the stressful situation (e.g., humor) (Lazarus and Folkman, 1984; Baqutayan, 2015).

In this regard, people who have experienced high levels of stress during the pandemic have reported engaging in coping activities such as physical activity or exercise, activities of spiritual or religious nature, seeking social support, talking about their worries and thinking in a positive way (Fu et al., 2020; Shechter et al., 2020). Regarding lockdown, some of the coping strategies that people mentioned were watching television, social networking, listening to music, sleeping, performing house chores, eating, or addressing piled-up work (Sameer et al., 2020).

Some of these simple coping strategies appear to protect against mental health problems. Thus, problem-focused strategies, including healthy habits such as following a healthy diet (Fullana et al., 2020), exercising regularly (Becerra-García et al., 2020; Lades et al., 2020; Parrado-González and León-Jariego, 2020; Yildirim and Güler, 2020), maintaining a routine in daily activities (Fullana et al., 2020; Parrado-González and León-Jariego, 2020), spending more time resting and relaxing (Zhang and Ma, 2020), and pursuing a hobby (Fullana et al., 2020; Lades et al., 2020) have been associated with lower levels of anxiety, depression, somatization symptoms, emotional distress, and others. However, some people have developed a passive or negative coping characterized by engaging in unhealthy or compulsive behaviors (Fu et al., 2020; Shechter et al., 2020; Dores et al., 2021). For example, emotional eating during lockdown was related to higher levels of anxiety, depression, and lower levels of quality relationships and quality of life (Cecchetto et al., 2021). Similarly, habits of substance use during the quarantine period such as drinking alcohol and smoking have been linked to more serious psychological problems (Chen et al., 2020), although fortunately, the likelihood of health risk behaviors decreased during the Spanish COVID-19 confinement (López-Bueno et al., 2020). Finally, the lack of effective strategies or problem-focused coping is related to mental health problems such as anxiety and sadness (Man et al., 2020).

On the other hand, emotion-focused strategies such as humor and positive reappraisal of COVID-19 experiences might be effective strategies. Research has shown that humor contributes to buffering the effects of negative or traumatic experiences through reappraisal and reinterpretation of negative events, generating a positive physiological effect and increasing social bonding (Kuyper et al., 2004; Martin, 2007; Sliter et al., 2013; Berk, 2015; Fritz, 2017). Thus, humor can promote positive affect, greater self-esteem, and well-being (Galloway and Cropley, 1999; Kuyper et al., 2004; Martin, 2007). Recent studies conducted during the pandemic have also revealed that humor is associated with lower levels of anxiety and fear of COVID-19 and better mental health (Eden et al., 2020; Saricali et al., 2020; Savitsky et al., 2020).

In summary, although there is considerable scientific evidence on the negative impact of COVID-19-derived confinement on mental health, the approach of this study is innovative for several reasons. Despite lockdown measures have been implemented in other countries, Spain may be considered a special case; the particular severity and duration of the stay-at-home quarantine that its citizens had to deal with, as well as the specificity of the Spanish culture (i.e., very sociable way of life) may have contributed to a harsher experience. Likewise, the characteristics of the instrument add specificity and novelty to the study. Concretely, the study uses the MHI-5 as a measure of well-being and distress. It is a mental health screening instrument especially recommended for use in the general population, rather than for psychiatric diagnostic purposes (Rivera-Riquelme et al., 2019). Most of the studies on mental health during lockdown have used psychiatric assessment/diagnostic instruments, so our study provides useful complementary data. In addition, considering the global concern about the mental health outcomes of COVID-19 pandemic, it is important to understand the effects of the lockdown in order to develop strategies and interventions for mental health, especially for high-risk groups. In light of this, the objective of our study is to identify factors that contributed to mental health during the lockdown decreed in Spain.

## Methods

### Participants and Data Collection

The sample was comprised of 3,508 participants from Spain ranging from 18 to 84 years old, with a mean age of 37.58 (*SD* = 15.8). By age range, 34.5% were 18–25 years old; 15.5% were aged 26–35; 25.6% were aged 36–50; 19.1% were aged 51–65 and 5.3% were 66 or older. Most were female (*n* = 2,496; 71%) and had completed university studies (*n* = 2,782; 79.3%).

After obtaining ethical and data protection approval, an online survey was designed using Google Forms. A link to the survey was sent via email and WhatsApp to multiple contacts, as well as posted on Facebook and Twitter. Participants were recruited via virtual snowball sampling, as they were asked to forward the link to their contacts. Informed consent was obtained by a compulsory question at the beginning of the survey. Data were collected during the phase of lockdown that took place in the months of March (*n* = 1,268; 35.1%), April (*n* = 747; 21.3%) and May (*n* =1,493; 42.6%) 2020.

### Measures

An online survey was applied. It included some questions designed *ad hoc*:

- Sociodemographics: Questions were asked regarding gender, age, educational level, employment status, economic stability, people living in the household (number and type of relationship), area of residence (rural vs. urban), country of residence.
- Daily planning and activity: Questions were asked regarding the activities carried out during the confinement period, the use (or not) of a daily schedule, number of days of confinement (at the time of answering the survey), number of outings during this period and the reasons for the outings.
- Healthy habits: another set of questions asked about the practice of healthy and unhealthy habits (healthy diet, exercise, smoking, drinking alcohol or taking sleeping pills).

The survey also included three standardized scales related to the following variables:

- Feelings of loneliness: The Spanish adaptation (Yárnoz, 2008) of the Social and Emotional Loneliness Scale for Adults-Short version (SESLA-S; DiTommaso et al., 2004) was used to assess the subjective experience of loneliness in adults in its three dimensions: Social Loneliness (e.g., I feel part of a group of friends), Family Loneliness (e.g., I feel close to my family) and Romantic/couple loneliness (e.g., I have a romantic or marital partner who gives me the support and encouragement I need). The three subscales have shown high internal consistency, and construct validity has been evidenced by significant correlations with measures of attachment style, psychological well-being and social desirability (Yárnoz, 2008). Responses were scored using a 4-point Likert-type scale ranging from 1 (*strongly disagree*) to 4 (*strongly agree*). Although the psychometric values of the scales are presented when discussing the measurement model of our data, the Cronbach's alpha value of this scale was, 86.
- Coping humor: The Spanish version (Caycho-Rodríguez et al., 2019) of the Coping Humor Scale (CHS-5) is a self-report measure that evaluates the “degree to which respondents make use of humor in coping with stress in their lives” (Martin, 1996, p. 251). The scale shows adequate reliability and validity according to the authors and is composed of five items (see Table 1). Responses were scored using a 4-point Likert-type scale ranging from 1 (*strongly disagree*) to 4 (*strongly agree*). The Cronbach's alpha value of the scale was 0.78 and the rho_A was 0.78 (Table 1).
- Mental Health: The Spanish version (Rivera-Riquelme et al., 2019) of the Mental Health Inventory-5 (MHI-5; Berwick et al., 1991) was used to screen psychological well-being and distress in the adult general population. Its psychometric coefficients indicate a good internal consistency. Responses were scored using a 5-point Likert-type scale ranging from 1 (*never*) to 5 (*almost always*). The five items are shown in Table 1. The Cronbach's alpha value, with our data, is 0.86 and the rho_A is 0.87 (Table 1).

**Table 1:** Evaluation of the assessment instrument.

	**Item**	**Outer Loading**	**α Cronbach**	**Rho_A**	**Composite Reliability**	**AVE**
Mental health	How much time, during the last month… …have you been a very nervous person? (recoded)	0.79	0.86	0.87	0.90	0.65
	.have you felt calm and peaceful?	0.80				
	.have you felt downhearted and blue? (recoded)	0.85				
	.have you been a happy person?	0.76				
	.have you felt so down in the dumps that nothing could cheer you up? (recoded)	0.81				
Healthy habits	Healthy diet (no excessive fat or sugar)	0.90	0.74	0.74	0.81	0.68
	Physical exercise (daily)	0.73				
Coping humor	I have often found that my problems have been greatly reduced when I have tried to find something funny in them	0.69	0.78	0.78	0.86	0.60
	I have often felt that if I am in a situation where I have to either cry or laugh, it's better to laugh	0.79				
	I can usually find something to laugh or joke about even in crying situations	0.81				
	It has been my experience that humor is often a very effective way of coping with problems	0.82				
Feelings of loneliness	Level of perceived loneliness	1^*†*^	1	1	1	1
People living in the household	How many people, including you, are currently living in your household?	1	1	1	1	1
Days of confinement	How many days have you been in confinement?	1	1	1	1	1
Area of residence	Where do you live during the confinement? Rural area (1); Urban area (2)	1	1	1	1	1
Gender	Male (1); Female (2)	1	1	1	1	1
Age	Age	1	1	1	1	1

† *For variables with only one indicator the value obtained is always 1*.

### Data Analysis

Data analyses were performed using Structural Equation Modeling (SEM) based on the analysis of variance (PLS; partial least squares). This statistical-mathematical procedure, currently known as second *generation multivariate analysis* (Hair et al., 2017), is rigorous and robust but also flexible because it does not impose strict assumptions about the distribution of variables, measurement scales, or sample size (Martínez and Fierro, 2018).

The PLS-SEM approach considers two levels of analysis: (1) structural model (*inner model*), and (2) measurement model (*outer model*). The latter establishes the quality and the contribution of each item to the measurement of the specific construct, that is, the relationships between the constructs (latent variables) and their indicators (observed variables), as well as the psychometric characteristics of these relationships. In turn, the structural model describes the relationships between the latent variables, i.e., the relationship between independent/exogenous variables and dependent/endogenous variables (Martínez and Fierro, 2018). This methodology is especially appropriate when the main objective is causal-predictive analysis, the research questions are complex, or the theoretical knowledge of the problem is scarce (Lévy and Varela, 2006). Smart-PLS version 3.3 (Ringle et al., 2015) was used to perform the data analysis. Statistical significance was set at *p* ≤ 0.05.

## Results

Table 2 presents the descriptive statistics and correlations of the investigated variables for the overall sample.

**Table 2:** Descriptive and correlational analysis of the model's numerical variables.

	**Mean**	**SD**	**Correlations**						
			**1**	**2**	**3**	**4**	**5**	**6**	**7**
1 Coping humor	12.81	3.66	1.00						
2 Healthy habits	5.09	1.41	0.132^**^	1.00					
3 Age	36.88	15.20	0.108^**^	0.051^**^	1.00				
4 People living in the household	3.09	1.55	−0.073^**^	−0.05	−0.240^**^	1.00			
5 Mental health	19.82	5.32	0.294^**^	0.215^**^	0.298^**^	−0.106^**^	1.00		
6 Feelings of loneliness	19.44	5.75	−0.116^**^	−0.169^**^	−0.147^**^	0.00	−0.362^**^	1.00	
7 Days of confinement	32.40	22.38	−0.235^**^	0.01	−0.255^**^	0.059^**^	−0.142^**^	0.03	1.00

** *The correlation is significant at the 0.01 level (bilateral)*.

Although the evaluation of the structural model and the measurement model is the starting point of the PLS-SEM methodology, the current tendency is to first analyze the validity of the overall model (Henseler et al., 2016a). “To test the fit of the model, one of the criteria used was the standardized root mean square residual (SRMR) normalization values which are values equal to or <0.06 indicate that a model is correctly specified (Henseler et al., 2016b). Other criteria used to evaluate model fit were the weighted least squares discrepancy (dULS) and the geodesic discrepancy (dG), values of which must be lower than the 95% quantile of their distributions (Henseler et al., 2016a).” According to these criteria, all the indicators of the proposed model showed good fit values (see Table 3).

**Table 3:** Goodness of fit indicators.

	**Saturated Model**	**Estimated Model**
SRMR	0.06	0.06
dULS	0.54	0.54
dG	0.18	0.18

### Measurement Model

As noted above, the measurement model specifies the relations between each latent variable and its indicators, which allows the evaluation of the quality of the measures involved in the overall model (Chen and Chang, 2011; Bernal et al., 2016).

The assessment of the measurement model based on reflective indicators should include the calculation of: (1) internal consistency reliability; (2) convergent validity (Average Variance Extracted [AVE] and indicators' reliability); and (3) discriminant validity (Hair et al., 2017). Internal consistency reliability was assessed using Cronbach's alpha and the composite reliability of the latent variables. The latter is the most adequate indicator when assessing PLS models (Chin, 1998), and values equal to or >0.70 indicate good internal consistency (Nunnally and Bernstein, 1994). Convergent validity refers to the extent to which the measures explain the variance of a construct and can be measured by the Average Variance Extracted. Values equal to or >0.50 are considered acceptable (Fornell and Larcker, 1981). The indicators' reliability or the simple correlations of each indicator with the construct can be measured by calculating the outer loadings. When interpreting, values equal to or >0.70 are considered adequate, while indicators with lower values should be removed. Table 1 includes only those indicators with admissible values.

Discriminant validity (Table 4) indicates the extent to which a construct differs from the others in the proposed model. As recommended by Henseler et al. (2015), the heterotrait-monotrait ratio of correlations (HTMT) was used to assess discriminant validity, since it is the best method to detect the absence of convergent validity. HTMT ratios must be <1 (Gold et al., 2001), as in the case of our model.

**Table 4:** HTMT ratios for the measurement model.

	**1**	**2**	**3**	**4**	**5**	**6**	**7**	**8**	**9**
1 Coping humor	-								
2 Healthy habits	0.23								
3 Age	0.14	0.13							
4 Gender	0.14	0.01	0.09						
5 People in the household	0.06	0.03	0.16	0.00					
6 Area of residence	0.03	0.08	0.09	0.00	0.10				
7 Mental health	0.40	0.32	0.32	0.19	0.08	0.02			
8 Loneliness	0.16	0.23	0.15	0.03	0.18	0.01	0.39		
9 Days of confinement	0.27	0.09	0.25	0.03	0.03	0.03	0.15	0.03	-

### Structural Model

The structural model is evaluated through four indicators: (1) *R*^2^, the coefficients of determination; (2) *Q*^2^, the predictive relevance of the model; (3) the significance of path coefficients; and (4) *f* ^2^, effect size.

**Figure 1:**
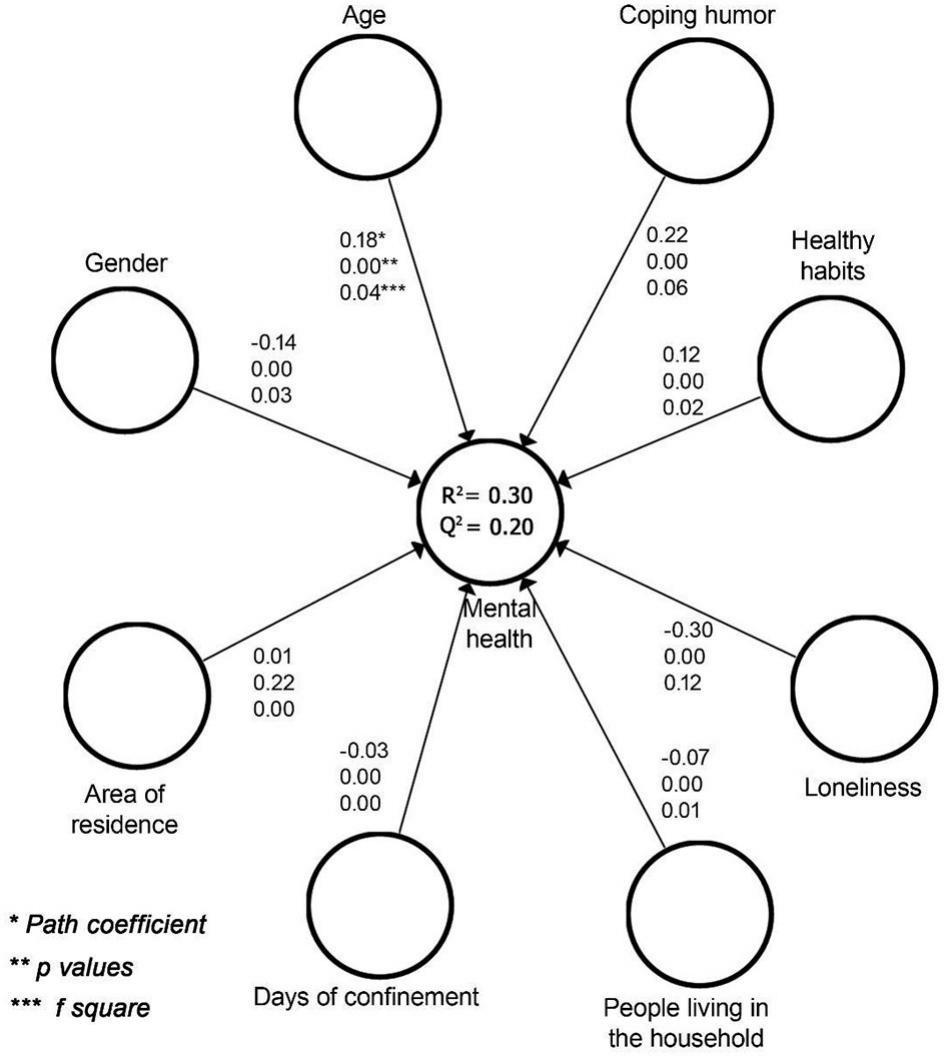
Values of the structural model.

*R*^2^ values allow the estimation of the predictive ability of the exogenous variables on the endogenous variables. According to the results (Figure 1), 30% of the variance in mental health was explained by latent variables. The values of these coefficients must be larger than 0.10; values of 0.25 or lower are considered significant but weak; values close to 0.40 are considered moderate; and values of 0.75 and above are considered large effects (Falk and Miller, 1992).

As a complement to *R*^2^, and in order to assess the predictive relevance of the model, it is also recommended to calculate the Cross-validated Redundancy (*Q*^2^) (Hair et al., 2017). These authors consider that values under 0.02 indicate a small fit; values close to 0.15 are considered a medium fit, and values of 0.35 and above indicate that the model has a high predictive validity. These *Q*^2^ values produced by the Stone-Giesser test (Geisser, 1974) are used as a criterion to measure the predictive relevance of the dependent constructs and is therefore a means to assess the predictive relevance of the structural model. If *Q*^2^ is >0 this implies that the model has predictive relevance (Hair et al., 2017). The statistical significance of the weights (*β*) is obtained by executing a bootstrapping procedure and from the Student's *t*-values and their corresponding significance levels. In our results, the weights of the different indicators were statistically significant, except for the area of residence (Figure 1). Moreover, the effect sizes were not statistically significant for two variables: number of days of confinement and number of people in the household.

*Loneliness* is the exogenous variable with the highest path coefficient as a predictor of mental health (*β* = −0.30; *p* < 0.05; *f* ^2^ = 0.12). Results showed an inverse relationship: as loneliness levels increased, levels of mental health decreased. The effect size allows us to conclude that loneliness was the latent variable that best predicted the dependent variable. The variable with the second highest factorial weight in the explanation of mental health was *coping humor* (*β* = 0.22; *p* < 0.05; *f* ^2^ = 0.06). The relationship between the variables was positive: as use of humor as a coping strategy increased, levels of mental health increased. Likewise, *healthy habits* showed a direct and statistically significant relation with mental health (*β* = 0.12; *p* < 0.05; *f* ^2^ = 0.02).

Among sociodemographic factors, *age* had a significant weight in the prediction of mental health during the pandemic (*β* = 0.18; *p* < 0.05). Considering that the relationship was positive, it may be concluded that the probability of psychological problems was higher among younger participants; the effect size and its significance corroborate the pertinence of this latent variable as a predictor (*f* ^2^ = 0.04). In addition, results confirmed an inverse and significant relation between *gender* and mental health (*β* = −0.14; *p* < 0.05). Given that arbitrary values of 1 and 2 were assigned to men and women, respectively, the data reveal that females are more likely than males to suffer mental health problems during the pandemic. As in the case of age, the significant effect size indicated that gender was a significant predictor of mental health (*f* ^2^ = 0.03).

By contrast, *area of residence* did not significantly relate to mental health (*β* = 0.01; *p* > 0.05; *f* ^2^ = 0.00). Similarly, neither length of confinement (*f* ^2^ = 0.00) nor number of people in the household (*f* ^2^ = 0.01) were significant predictors, although their weights were statistically significant. Specifically, the number of *people in the household* was positively related (*β* = −0.07; *p* < 0.05), and the number of *days of confinement* was negatively related to mental health (*β* = −0.03; *p* < 0.05).

## Discussion

Mental health is a complex construct that is determined by many individual, social and societal risk and protective factors, and by all the interactions of these factors (Sturgeon, 2007). The aim of this study was to identify factors that contributed to mental health during the lockdown decreed in Spain as a consequence of the coronavirus crisis.

Of the factors analyzed, loneliness seems to be a particularly important risk factor for mental health, as indicated by previous literature (e.g., de Jong Gierveld, 1998; Patterson and Veenstra, 2010; Steptoe et al., 2013; Richard et al., 2017). Before the pandemic crisis, loneliness was already a public health concern (Richard et al., 2017; Cacioppo and Cacioppo, 2018; Ercole and Parr, 2020), but social distancing and lockdown measures taken to control the spread of COVID-19 could potentially increase feelings of loneliness, especially among people at risk for social isolation or chronic medical conditions (Killgore et al., 2020; Luchetti et al., 2020). Furthermore, a study by Robb et al. (2020) found a negative relationship between loneliness and mental health (depression and anxiety) following the lockdown in London. In addition, these results are consistent with literature that demonstrates the relationship of social support and positive connection with family members and friends with mental health (Turner and Brown, 2010; Wang et al., 2018) and positive adaptation to the COVID-19 pandemic (Saltzman et al., 2020).

Likewise, the ability to cope with humor during the pandemic and lockdown seems to predict better mental health. Humor has psychological and physiological benefits, especially for older adults (Berk, 2015). When used as a coping strategy, it may help to deal with adversity by moderating emotions and releasing tension, primarily when it is used in social contexts and shared with others (Martin, 2007). As previous studies showed, humor may serve as a resilience factor during lockdown and during the pandemic, by permitting modification of negative explanations about COVID-19 (Eden et al., 2020; Saricali et al., 2020; Savitsky et al., 2020) and by inducing positive emotional states and connection with other people through sharing jokes and laughs. Similarly, and in line with prior research (e.g., Becerra-García et al., 2020; Lades et al., 2020; Parrado-González and León-Jariego, 2020), this study suggests that both regular physical exercise and healthy diets partly influence mental health during a pandemic, specifically acting as protective factors. However, caution should be taken as the effect sizes for these variables, although significant, were small in this study.

With regard to the influence of sociodemographic factors, it appears that the likelihood of psychological problems during a pandemic is higher among younger people, in line with other studies in Spain (Becerra-García et al., 2020; Caballero-Domínguez et al., 2020; González-Sanguino et al., 2020; Parrado-González and León-Jariego, 2020; Rodríguez-Rey et al., 2020) and abroad. Increased vulnerability was also observed among children and adolescents in the context of COVID-19 (Loades et al., 2020). Possible explanations for this finding may be that the ability to psychologically self-regulate increases with age (Wang et al., 2021) or that old adults could have resources that increase their resilience (López et al., 2020). Younger people may also have greater difficulties dealing with social isolation and, in the case of students, with an atypical academic year (Parrado-González and León-Jariego, 2020). This result may help explain the risky behaviors of some young people throughout the pandemic. For instance, the symptomatology of anxiety and depression may foster involvement in high-risk situations (e.g., going out without safety measures, attending crowded parties), combined with other characteristics of this age group (e.g., low risk perception, higher likelihood of recovering from the disease without serious health problems).

Women also appear to be more likely to suffer from mental health symptoms or problems due to the pandemic, in accordance with prior results (e.g., Barzilay et al., 2020; González-Sanguino et al., 2020; Parrado-González and León-Jariego, 2020; Rodríguez-Rey et al., 2020; Xiong et al., 2020; Dores et al., 2021). These gender differences have been observed in epidemiological studies with the general population (Lim et al., 2018) and theoretically linked to females' greater vulnerability to stressful events, their greater tendency to verbalize their distress (Parrado-González and León-Jariego, 2020) or their social role as the primary caregiver of the family (Wang et al., 2021) and the old adults. As research by Farré et al. (2020) points out, during the Spanish lockdown, “women were more likely to be furloughed, unemployed or working from home, and took on most of the childcare and household chores” (p. 20). This overload of work and responsibilities could contribute to explaining the gender differences in mental health. Greater attention to the specific challenges and experiences faced both by women and by youth during the lockdown is warranted.

This study also sheds light on the non-significant sociodemographic factors. Thus, similarly to previous data (Lu et al., 2020) but contrary to other results (El-Zoghby et al., 2020), living in a rural environment or in a city does not appear to be relevant as a predictor of mental health, at least in this period of confinement; furthermore, given its non-significant effect size, this variable should be eliminated from the final mental health predictive model. Similarly, although they obtained significant weights, the two variables of time spent confined and number of cohabitants in the household should be eliminated from the final predictive model due to their non-significant predictive capacity. Therefore, in line with previous results (e.g., Twenge and Joiner, 2020), it does not appear true that the longer the confinement lasted, the more likely the mental health of the general public was seriously affected (at least in Spain), maybe as a consequence of adaptation and/or resignation, or perhaps because being confined increases the perception of safety and helps reduce the fear of disease transmission.

As regards limitations and future directions, the obtained sample may not be representative of the general Spanish population due to the use of a snowball sampling technique and the implementation of an online survey. For example, as in other studies (e.g., González-Sanguino et al., 2020), older adults aged 65 or above were under-represented, so caution should be taken when extrapolating results to this age group. In addition, the procedure and methodology employed in the study hindered the collection of data from those with limited access to technology and the internet, who might experience higher levels of loneliness and social disconnection and consequently present lower levels of mental health. Future studies should consider administering a survey by phone or via the postal service and examine the role that the interactions of some variables (e.g., age and humor) or combination of variables could have on mental health levels. The cross-sectional nature of the data does not allow clear identification of the effects on mental health of Spanish people during the lockdown period. Although the model presents adequate values of fit, we must be cautious in the interpretation of some of its values, even if they are statistically significant, due to the complexity of the intervening variables and the nature of mental health. It should be noted that coping strategies and healthy lifestyle habits are among the lesser significant variables, showing significant values of path coefficient but small values of variance explained of mental health (*f* ^2^). Finally, this study has detected significant relationships between different variables, such as age and loneliness. Given that the data analysis carried out in this article does not allow to explore these and other significant relationships in depth, it is considered as a future line of research.

## Conclusions

The present study contributes to addressing the effect of COVID-19 and strict lockdown measures by identifying risk factors for the development of mental health difficulties. When interpreting and extrapolating our findings, researchers should bear in mind Spain's uniqueness and commonality as regards lockdown severity and social culture. For example, our conclusions might be more generalizable to countries with more similar stay-at-home restrictions and/or cultural characteristics (e.g., Italy, Israel), but perhaps less applicable to other countries that adopted milder lockdown measures during the COVID-19 first wave (e.g., Norway) or which are culturally different in terms of social interactions and physical distance (e.g., United Kingdom).

Both clinical and public health implications can be drawn. First, as other authors have pointed out (e.g., Brooks et al., 2020; Roychowdhury, 2020), restrictive measures may be necessary during the pandemic, but they must be handled with caution and providing citizens with resources to face them, as they are not free of risks and challenges. Thus, mental health should be included in public health emergency programs. Policymakers and health care providers should pay attention not only to the impact of the pandemic on physical health, but also consider mental health outcomes, addressing psychiatric symptomatology as well as other mental health difficulties such as distress that can remain hidden and gradually erode the psychological resources of citizens, thus reducing their well-being if maintained over time. Now that we know the possible consequences of severe confinement measures, we can begin to design plans and strategies to control the spread of the virus and prevent mental health problems in the population.

Second, our findings add evidence to previous studies suggesting that the psychological impact of lockdown depends on multiple factors such as gender, age, loneliness, healthy habits and coping humor. This evidence may help in the detection of particularly vulnerable individuals and allow the targeting of specific and early interventions aimed at reducing risk factors and fostering protective factors to mitigate the impact on mental health outcomes and increase the well-being of the population. Thus, our findings suggest that there is a need to reinforce social support services for women and youth and facilitate access to mental health care. Again, it is essential that public health make efforts to meet the psychosocial needs of the population, both during the pandemic and in the long term.

Third, it is particularly important to consider which coping strategies, positive traits and resilience resources could improve personal growth, adjustment and mental health during lockdown (López et al., 2020). Lessons learned during the pandemic should help in the design of targeted interventions to alleviate the perception of loneliness (e.g., virtual support groups or talk therapies) and provide psychoeducation about effective coping strategies and healthy habits.

## Data Availability Statement

The raw data supporting the conclusions of this article will be made available by the authors, without undue reservation.

## Ethics Statement

The studies involving human participants were reviewed and approved by the Research Ethics Committee of the University of Valladolid and the Ethics Committee of the Regional Sanitary System (SACYL). The patients/participants provided their written informed consent to participate in this study.

## Author Contributions

RP and AP contributed to conception and design of the study. All authors contributed to the collection of the data. AP organized the database and performed the statistical analysis. IV-M and EG wrote the first draft of the manuscript. All authors contributed to the manuscript revision, read, and approved the submitted version.

## Conflict of Interest

The authors declare that the research was conducted in the absence of any commercial or financial relationships that could be construed as a potential conflict of interest.

## Publisher's Note

All claims expressed in this article are solely those of the authors and do not necessarily represent those of their affiliated organizations, or those of the publisher, the editors and the reviewers. Any product that may be evaluated in this article, or claim that may be made by its manufacturer, is not guaranteed or endorsed by the publisher.
